# Two-Component-System RspA1/A2-Dependent Regulation on Primary Metabolism in *Streptomyces albus* A30 Cultivated With Glutamate as the Sole Nitrogen Source

**DOI:** 10.3389/fmicb.2020.01658

**Published:** 2020-07-31

**Authors:** Kuipu Zhang, Ali Mohsin, Junxiong Yu, Yuwen Hu, Muhammad Fahad Ali, Zhongbing Chen, Yingping Zhuang, Ju Chu, Meijin Guo

**Affiliations:** ^1^State Key Laboratory of Bioreactor Engineering, East China University of Science and Technology, Shanghai, China; ^2^College of Food Science and Technology, Nanchang University, Nanchang, China; ^3^Zhejiang Biok Biology Co., Ltd., Zhejiang, China

**Keywords:** two-component system, phosphoenolpyruvate-pyruvate-oxaloacetate node, gluconeogenesis, *Streptomyces albus*, glucose consumption rate, transcriptome analysis

## Abstract

In our previous study, a two-component-system (TCS) RspA1/A2 was identified and proven to play a positive role in the regulation of salinomycin (antibiotic) biosynthesis in *Streptomyces albus*. However, the regulatory mechanism of RspA1/A2 using a carbon source (glucose or acetate) for the cell growth of *S. albus* is still unclear till present research work. Therefore, in this work, the mechanistic pathway of RspA1/A2 on carbon source metabolism is unveiled. Firstly, this work reports that the response regulator RspA1 gene *rspA1* knocked-out mutant ΔrspA1 exhibits lower biomass accumulation and lower glucose consumption rates as compared to the parental strain A30 when cultivated in a defined minimal medium (MM) complemented with 75 mM glutamate. Further, it is demonstrated that the regulation of TCS RspA1/A2 on the phosphoenolpyruvate-pyruvate-oxaloacetate node results in decreasing the intracellular acetyl-CoA pool in mutant ΔrspA1. Subsequently, it was verified that the RspA1 could not only directly interact with the promoter regions of key genes encoding AMP-forming acetyl-CoA synthase (ACS), citrate synthase (CS), and pyruvate dehydrogenase complex (PDH) but also bind promoter regions of the genes *pyc*, *pck*, and *glpX* in gluconeogenesis. In addition, the transcriptomic data analysis showed that pyruvate and glutamate transformations supported robust TCS RspA1/A2-dependent regulation of glucose metabolism, which led to a decreased flux of pyruvate into the TCA cycle and an increased flux of gluconeogenesis pathway in mutant ΔrspA1. Finally, a new transcriptional regulatory network of TCS RspA1/A2 on primary metabolism across central carbon metabolic pathways including the glycolysis pathway, TCA cycle, and gluconeogenesis pathway is proposed.

## Introduction

Generally, in many bacteria, the utilization of carbon sources in cells via oxidative reactions to generate reducing equivalents (NADH, NADPH), energy source ATP, and intermediate metabolites is considered as a complex and precisely regulated process. Some key genes located in the central carbon metabolic pathways play crucial roles in regulating the flux of glycolysis and TCA cycle. For example, *pdh* encodes pyruvate dehydrogenase complex (PDH), which serves as a housekeeping enzyme for energy metabolism by catalyzing the irreversible decarboxylation of pyruvate into acetyl-CoA and is deemed the key control point in glycolysis (Holness and Sugden, 2003; Tomar et al., 2003; Guest et al., 2006). In both prokaryotic and eukaryotic organisms, the activity of PDH is regulated by various metabolites like acetyl-CoA, oxaloacetate, and ATP (Patnaik and Liao, 1994; Commichau et al., 2013). For example, *Escherichia coli* strains with mutations in genes encoding the subunits E1 or E2 (*aceE* and *aceF*, respectively) of the PDH exhibited significant pyruvate accumulation and growth defect (Tomar et al., 2003). Apart from PDH, citrate synthase (CS) was also a potential control point in affecting the flux of the glycolysis pathway and TCA cycle. Therefore, CS appeared to be strongly regulated in microorganisms. For instance, the *gltA*, as a single CS gene in *E. coli*, was repressed by the ArcA two-component response regulator in response to anaerobiosis and changes in carbon source supply (Iuchi and Lin, 1988). The *citZ* gene encoding CS in the gram-positive bacterium *Bacillus subtilis* was controlled by three regulatory proteins, CodY, CcpA, and CcpC (Sonenshein, 2007).

Moreover, under gluconeogenic conditions, like a limited carbon source or sufficient nitrogen source medium, the TCA cycle intermediates like oxaloacetate (OAA) or malate are converted into pyruvate and PEP by decarboxylation (C4-decarboxylation) under the catalysis of PEP carboxykinase (PCK) or malic enzyme (MAE) (Utter, 1972; Hansen and Juni, 1974). Hence, the intermediates of the PEP-pyruvate-oxaloacetate node provide direct precursors for gluconeogenesis. Additionally, the PEP-pyruvate-oxaloacetate node is located in the metabolic link between glycolysis/gluconeogenesis and the TCA cycle, which acts as a highly central control point for glucose consumption and glucose-generating metabolism. The regulation of carbon flux at the PEP-pyruvate-oxaloacetate node is mainly focused on the enzymes at the PEP-pyruvate-oxaloacetate node in bacteria. This kind of regulation is usually subjected to a complex allosteric regulation, in which the commonly used effectors are NADH, acetyl-CoA, NADPH, energy charge, ADP, and AMP. For instance, in previous studies, PEP synthase was repressed by ADP and AMP, whereas it was activated by energy charge (Chulavatnatol and Atkinson, 1973), however, PEP carboxykinase was repressed by ATP and PEP (Ken-ichi et al., 2001; Blencke et al., 2003; Servant et al., 2005). Furthermore, acetyl-CoA and long-chain fatty acids were reported as positive effectors of PEP carboxylase (Silverstein and Willis, 1973).

Most importantly, there exists a complex transcriptional regulatory network, which is involved in the primary metabolism of *Streptomyces*. One of the best-studied regulators related to carbon metabolism in the *Streptomyces* is DasR, a GntR-family transcriptional factor (Romero-Rodríguez et al., 2015). DasR revealed its direct regulation of the genes encoding citrate synthase in *Saccharopolyspora erythraea* and its N-acetylglucosamine-6-phosphate (GlcNAc-6P)-dependent influences on acetate metabolism in *S. erythraea* and *S. coelicolor* (Seo et al., 2002; Liao et al., 2014). GlnR, initially demonstrated for its regulation of nitrogen source metabolism in *Streptomyces* (Antonio et al., 2009; Wang and Zhao, 2009), was also proven to directly control the transcription of the ATP-binding cassette (ABC) transporter in Actinomycetes, which affects the uptake of carbon source (Liao et al., 2015). Moreover, several genes at the PEP-pyruvate-oxaloacetate node were known as the targets of global transcriptional regulators. *pckA*, for example, was repressed in a Crp-dependent manner, and repression was relieved at low cAMP levels (Gosset et al., 2004). Besides that, *pckA* was also induced by the catabolite repressor/activator Cra (also known as FruR) (Saier and Ramseier, 1996).

Salinomycin, being one of the important antibiotics biosynthesized by *S. albus*, has been widely utilized in agriculture as the treatment of coccidiosis for its ability to inhibit the growth of majority of gram-positive bacteria (Gumila et al., 1997). SlnR was proven as a positive pathway-specific regulator for salinomycin biosynthesis and could modulate the transcription of genes in the salinomycin biosynthetic cluster in *S. albus* (Zhu et al., 2017). In our previous study, a two-component-system (TCS) RspA1/A2 (*slnwt_4828/4829*), which is homologous to TCS AfsQ1/Q2 in *S. coelicolor*, was proven to be a positive regulator for salinomycin biosynthesis by directly binding the promoter region of the gene *slnR* in *S. albus* when grown under the condition of a YMG medium supplemented with 75 mM glutamate (unpublished work). Previously, TCS AfsQ1/Q2 was firstly reported to promote the biosynthesis of actinorhodin (ACT) and undecylprodigiosin (RED) in *Streptomyces lividans* (Ishizuka et al., 1992). In 2009, Shu et al., further demonstrated that the *afs*Q1 mutant derived from *S. coelicolor* A3(2) exhibited significantly decreased ACT, RED, and calcium-dependent antibiotic (CDA) production when cultivated on a defined minimal medium (MM) with 75 mM glutamate as the sole nitrogen source (Shu et al., 2009). Interestingly, Wang et al. suggested that TCS AfsQ1/Q2 had potential significant influences on carbon metabolism in *S. coelicolor* (Wang et al., 2012). However, the regulatory mechanism of the TCS AfsQ1/Q2 in the primary metabolism of the carbon source in *Streptomyces* has not been elucidated clearly so far.

Herein, our main aim is to explore the regulatory mechanism of TCS RspA1/A2 that affects glucose consumption through central carbon metabolic pathways for cell growth in *S. albus* when cultivated in a minimal medium (MM) supplemented with glutamate as the sole nitrogen source. To the best of our knowledge, it is the first study so far to focus on exploring the transcriptional regulatory network of TCS RspA1/A2 on the glucose metabolic pathways in the genus *Streptomyces*. Furthermore, in this work, several new binding sites of the TCS response regulator RspA1 were identified as located in glycolysis/gluconeogenesis and the TCA cycle. Finally, the differences in glucose metabolic flux distribution between mutant ΔrspA1 and the initial strain A30 were compared based on a transcriptomic dataset.

## Materials and Methods

### Bacterial Strains and Growth Conditions

The parent strain A30 (Provided by Zhejiang Biok Biology Co., Ltd.) and its derivatives were grown on ISP4 agar plates (BD, United States) for sporulation. For the shaking flask fermentation, 150 μL of the parental strain A30 and its derivatives spore suspension (OD_450_ = 1.0) was inoculated into a 500 mL flask containing 100 mL of fresh fermentation medium (glucose 10 g/L, casamino acid 5.0 g/L, NaCl 2.0 g/L, KCl 2.0 g/L, K_2_HPO_4_ 0.2 g/L, MgSO_4_ 0.1 g/L, CaCl_2_ 0.1 g/L, CaCO_3_ 2 g/L, pH 7.0) supplemented with 75 mM glutamate, or minimal medium (MM) (Islam, 2009) in which ammonium was replaced by 75 mM L-glutamate (Glu) as the sole nitrogen source, or Evans medium [25 mM TES [N-(Tris(hydroxymethyl)methyl)22-aminoethanesulfonic acid sodium salt), 2 mM citric acid, 10 mM KCl, 0.25 mM CaCl_2_, 1.25 mM MgCl_2_, 2 mM Na_2_SO_4_, 1 mM Na_2_MoO_4_, 0.5% trace elements (0.02 mM MnSO_4_⋅4H_2_O, 6 μM ZnSO_4_⋅7H_2_O, 0.02 mM H_3_BO_3_, 1 μM KI, 2 μM Na_2_MoO_4_⋅2H_2_O, 0.05 mM CuSO_4_⋅5H_2_O, 0.05 mM CoCl_2_⋅6H_2_O, 20 g/L glucose, 2 mM NaH_2_PO_4_, pH 7.2] supplemented with 75 mM glutamate.

### Construction of *rspA1* Mutants

The parent strain A30, the gene *rspA1* knocked-out mutant ΔrspA1, and its complementary mutant ΔrspA1a were constructed in this study, and primers used in this study are shown in Supplementary Table S1. Gene amplification was based on the genomic sequence of *S. albus* DSM41398^1^. The separation between *rspA1* and *rspA2* is 36 bp, indicating that *rspA1* and *rspA2* were co-transcribed, so we replaced a 586 bp fragment (located within the deletion region of *rspA1* gene) with the kanamycin resistance gene to construct a *rspA1/A2* gene deletion mutant in A30; a *Bam*HI*/Hin*dIII fragment containing the kanamycin resistance gene *neo* was ligated with *Bam*HI*/Hin*dIII-digested pJTU1278. Then, a 3.8 kb *Bam*HI fragment of the left flanking region and a 4.0 kb *Hin*dIII fragment of the right flanking region were ligated with *Bam*HI- or *Hin*dIII-digested pJTU1278 to generate a plasmid for *rspA1* deletion, respectively. After that, the resulting plasmid was transferred into A30 by conjugation from *E. coli* ET12567/pUZ8002; the selected colonies were further confirmed by DNA sequencing. We thus obtained the *rspA1* gene deletion mutant ΔrspA1, in which the *rspA1* gene was completely deleted by double-crossover recombination.

For complementation and overexpression of *rspA1*, the whole CDS region of *rspA1* was amplified by PCR with primer pair *rspA1*-F-*Nde*I and *rspA1*-R-*Eco*RI, and the product was cloned to pMD18-T and verified via sequencing. An *Nde*I*-Eco*RI fragment with the *rspA1* gene was ligated with *Nde*I-*Eco*RI-digested pIB139 to generate pIB-*rspA1* (Bierman et al., 1992). Then, an *Eco*RI fragment containing the apramycin resistance gene *aacIV* was ligated with *Eco*RI-digested pIB-*rspA1*. The resulting plasmid was introduced into wild-type competent cells through conjugation, and apramycin-resistant exoconjugants were selected and further confirmed by PCR with corresponding PCR primers.

### Fermentation of Mutant ΔrspA1 and the Original Strain A30 in a 5 L Fermenter

The 150 μL amount of spore suspension (OD_450_ = 1.0) was inoculated into a TSBY liquid medium in a 250 mL flask and cultivated with shaking at 220 rpm on a rotary shaker at 34°C for 36 h. Then, 100 mL of the seed culture was centrifuged, and cell pellets were re-suspended in a 100 mL fermentation medium. After that, the resuspension was transferred into a 5 L bioreactor containing 3 L of fermentation medium with 20 g/L glucose. The initial pH was adjusted at 7.0 and dissolved oxygen (DO) tension at around 30% was controlled by agitation speed with a constant aeration rate of 1.0 VVM (air volume/medium volume per minute). The analysis of residual sugar and dry cell weight (DCW) was detected off-line by following standard methods. Whereas the parameters of oxygen and carbon dioxide contents in exhaust gas were analyzed on-line by mass spectrum (Thermo Scientific, Prima BT, United Kingdom), simultaneously, oxygen uptake rate (OUR) and carbon dioxide evolution rate (CER) were calculated at-line.

### RNA Extraction and RNA Sequencing

RNA samples from mutant ΔrspA1 and the parental strain A30 were analyzed at 30 h in a fermentation medium of shaking flask culture. After that, the samples were directly frozen in liquid nitrogen and stored at −80°C.

Sequencing and subsequent bioinformatics analysis were completed on Novel Bioinformatics Co., Ltd., Shanghai, China, as described previously (Zhang et al., 2019). Total RNAs were extracted with a trizol reagent (Invitrogen, Grand Island, NY, United States). The RNA quality was checked by capillary electrophoresis (Bioanalyzer 2200; Aligent, Santa Clara, CA, United States). Ribosomal RNA was depleted with Ribo-Zero^TM^ rRNA Removal Kits (Illumina Inc., San Diego, CA, United States), and the cDNA library construction was carried out using the TruSeq Stranded mRNA Library Preparation Kit (Illumina). Raw data are available on the Gene Expression Omnibus database (accession **GSE143602**).

### Overproduction and Purification of TCS Response Regulator RspA1

To express TCS response regulator RspA1, the open reading frame of *rspA1* gene was amplified by PCR from the genomic DNA of *S. albus* DSM 41398 (version: GCA_000827005.1). After digesting by restriction enzymes, gene *rspA1* encoding for RspA1 was inserted into plasmid pET28a then transformed into *E. coli* BL21 (DE3) competent cells to overexpress RspA1 protein. A single colony of transformant was picked into 5 mL of LB medium containing kanamycin and then grown at 37°C overnight. Then, cells were inoculated into 100 mL of LB medium supplemented with 50 mg/L kanamycin. 0.5 mM isopropyl-b-D-thiogalactoside (IPTG) was added when cells were grown to OD_600_ of 0.4–0.8, then the temperature was shifted to 20°C for 12 h.

Protein His-RspA1 was purified as described previously (You et al., 2017), the fractions were analyzed by SDS-PAGE and the protein concentration was determined by the BCA method using bovine serum albumin as a standard.

### Electrophoretic Mobility Shift Assay (EMSA)

The binding sites were supposed to be located on the upstream region (−300 to + 50 bp relative to transcription start site of target genes); primers were designed and attached with a universal primer (5′-AGCCAGTGGCGATAAG-3′) and are shown in Supplementary Table S1. The PCR products were biotin-labeled by PCR with a 5′ biotin-modified universal primer. Concentrations of PCR products were determined by NanoDrop 2000 (Thermo Fisher Scientific, Germany). EMSAs were carried out according to the protocol provided by Chemiluminescent EMSA Kit (Beyotime Biotechnology, China). The binding reaction consisted of 10 mM Tris-HCl pH 8.0, 25 mM MgCl_2_, 50 mM NaCl, 1 mM DTT, 1 mM EDTA, 0.01% Non-idet P40, 50 mg/L poly[d(I-C)], and 10% glycerol. After binding, the samples were separated on a non-denatured PAGE gel in ice-bath at 100 V for 90 min and transferred into an N^+^ nylon membrane (GE Amersham, United States). At last, the bands were detected by BeyoECL Plus after dyeing (Beyotime Biotechnology, China).

### RNA Preparation and Real-Time qRT-PCR

Samples were taken from *S. albus* cultures grown in a fermentation medium supplemented with 75 mM glutamate in shaking flask culture. RNA extractions were performed with an RNA extraction kit (Sangon Biotech Co., Ltd., Shanghai, China) by following the manufacturer’s instructions. To remove chromosomal DNA contamination, each RNA sample was treated with DNase I (Takara, Japan) for 5 min at 42°C and subsequently confirmed by PCR using different primer pairs (Supplementary Table S1). After that, the concentration of total RNA was determined by NanoDrop 2000 (Thermo Fisher Scientific, Germany). RNA samples (2 μg) from two biological replicates were then transcribed using a PrimeScript^TM^ RT Reagent Kit with gDNA Eraser (Takara, Shiga, Japan) for real-time RT-PCR. All these transcribed procedures mentioned above were performed as per the manufacturer’s instructions.

SYBR premix Ex Taq^TM^ GC Kit Perfect Real Time (Takara, Shiga, Japan) was used, and about 1 μL cDNA was added into 25 μL volume of PCR reaction for real-time RT-PCR. The PCR was conducted using CFX96 Real-Time System (Bio-Rad, United States) and the conditions were 95°C for 5 min, then 40 cycles of 95°C for 5 s, 58°C for 30 s, and an extension at 72°C for 10 min. For all the RT-PCR assays, 16S rRNA was used as an internal control. The relative fold changes of gene transcription were calculated by using the 2^–ΔΔ*Ct*^ method (Livak and Schmittgen, 2001). Quantitative real-time PCR (qRT-PCR) experiments were conducted with three independent biological replicates, and error bars indicate the standard deviations (SD).

Expression of the most differentially expressed genes (*slnwt_3899*, *pyc*; *slnwt_4964*, *pck*; *slnwt_2304*, *glpX*; *slnwt_5962*, *pgi*) between mutant ΔrspA1 and the initial strain A30 was validated by qRT-PCR. The *gap* gene (*slnwt_5957)* and genes encoding PDH (*acE*, *slnwt*_3555–*slnwt*_3557) were also analyzed. The correlation between transcript abundances quantified by RNA-seq and qRT-PCR was adequate (regression coefficient of 0.74) (Supplementary Figure S1).

## Results

### Mutation of *rspA1* Affects Biomass Accumulation and Glucose Consumption Rate in *Streptomyces albus*

**FIGURE 1:**
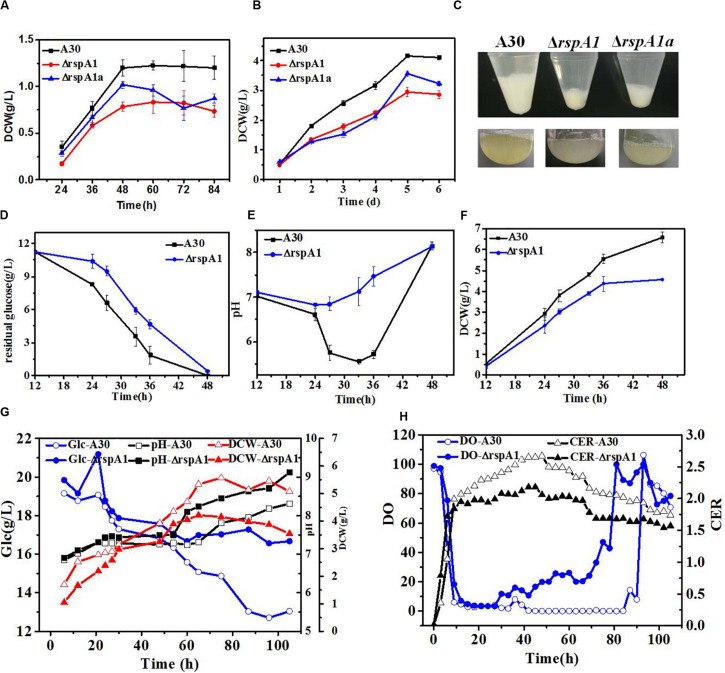
The mutation of *rspA1* affects biomass accumulation and glucose consumption rate in *Streptomyces albus* when cultured in different media. **(A,B)** Growth differences among mutant ΔrspA1, ΔrspA1a, and the initial strain A30 when cultured in an MM medium **(A)** or EVANS base **(B)** complemented with 75 mM sodium glutamate. **(C)** Pellets of a 3 mL fermentation broth after a 12 h culture. **(D–F)** Residual sugar, pH and dry cell weight (DCW) of mutant ΔrspA1, ΔrspA1a, and the initial strain A30 when cultured in a fermentation medium complemented with 75 mM sodium glutamate. **(G)** Parameters of residual sugar, pH, and DCW when cultured in a 5L bioreactor. **(H)** On-line parameters of dissolved oxygen (DO) and carbon dioxide evolution rate (CER) in exhaust gas when cultured in a 5 L bioreactor.

*S. albus rspA1* knocked-out mutant ΔrspA1 did not show any obvious variation in growth when cultured neither in TSB nor in ISP4 liquid medium (Supplementary Figures S2A,B). However, when the culture medium was replaced by MM or EVANS base medium complemented with 75 mM glutamate, mutant ΔrspA1 showed a lower biomass accumulation as compared to the original strain A30, whereas the impaired growth was partially restored in the complemented mutant ΔrspA1a (Figures 1A–C). Furthermore, we investigated the growth differences between mutant ΔrspA1 and the initial strain A30 when cultured in an MM solid plate complemented with various amino acids. No striking phenotypic differences were observed between mutant ΔrspA1 and the original strain A30 when cultured on an MM solid plate complemented with phenylalanine (Phe), histidine (His), and aspartate (Asp). However, the growth of the initial strain A30 was comparatively better than mutant ΔrspA1 when cultured in a medium with 75 mM glutamate (Supplementary Figure S3).

In order to further explore the effects of organic nitrogen and inorganic nitrogen sources on the cell growth of mutant ΔrspA1 and the original strain A30, the growth differences between mutant ΔrspA1 and the original strain A30 were compared when the nitrogen source in the fermentation medium of shaking flask culture was 5 g/L ammonium sulfate and 75 mM sodium glutamate, respectively. As shown in Supplementary Figures S2C–E, when 5 g/L ammonium sulfate was used as a nitrogen source, the trends of glucose consumption rate, pH, and DCW exhibited no significant differences between mutant ΔrspA1 and the original strain A30. However, when 75 mM glutamate was used as a nitrogen source, the sugar consumption rate of the original strain A30 was significantly faster than that of mutant ΔrspA1 within the first 33 h of culture time. With the decrease of glucose content, the sugar consumption rate of strain A30 started to slow down at 33 h. Although mutant ΔrspA1 consumed the same amount of carbon source as the original strain A30 during the entire culture course, the ultimate glucose consumption rate of mutant ΔrspA1 was smaller than the original strain A30 (Figure 1D). As a result, the original strain A30 accumulated more biomass, up to 6.6 g/L, which is 43% more than that of mutant ΔrspA1.

To verify differences in the physiological state between mutant ΔrspA1 and original strain A30, both of them were cultivated in a 5 L fermenter, respectively. As shown in Figures 1G,H, the profiles of residual sugar, pH, and DCW in a 5 L fermenter were consistent and almost the same as in shaking flask culture (Figures 1D–F). Generally, with regard to mutant ΔrspA1 fermentation, the DO started to increase when glucose was no longer consumed at around 40 h in fermentation broth, which is earlier than the initial strain A30 that occurred at around 80 h. Meanwhile, the CER of mutant ΔrspA1 was found lower than that of the initial strain A30 due to its slower glucose consumption, corresponding with the trend of residual sugar concentration (Figures 1G,H). Conclusively, the TCS response regulator gene *rspA1* was knocked out, resulting in the impaired cell growth and a smaller glucose consumption rate of mutant ΔrspA1 when glutamate was utilized as the sole nitrogen source in fermentation medium. As such, the transcriptional regulatory mechanism of TCS RspA1/A2 affecting glucose primary metabolism through central carbon metabolic pathways is further investigated in the following works.

### RspA1 Directly Activates the Transcription of *acsA* Gene

Using a previous training set developed for RspA1 binding-site (RspA1-Box) prediction (Wang et al., 2012), putative binding sites of the RspA1 were found in the upstream region of acs genes *slnwt_6888* and *slnwt_2998* (*slnwt_6888*: TGTTCGTAGGTGCCAC; *slnwt_2998*:CCTCCGATCCTGGCAC). Acetyl-CoA synthase is essential for cell growth in the MM medium, with acetate as the sole carbon source (You et al., 2017). Furthermore, Acetyl-CoA synthase catalyzes the synthesis of acetyl-CoA with acetate as the substrate, which is a crucial supply for acetyl-CoA. Therefore, Acetyl-CoA synthase could be a potential control point for TCS RspA1/A2.

**FIGURE 2:**
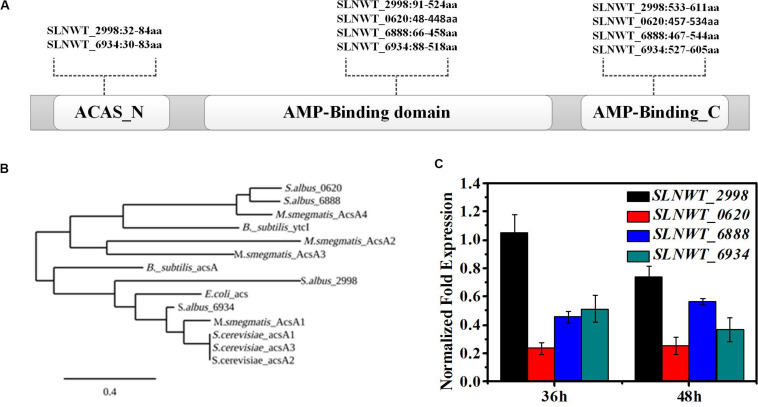
RspA1 directly controls the transcription of *acsA*. **(A)** Predicted domains of Acs are shown in differently colored boxes. **(B)** Phylogenetic analysis of ACSs of *S. albus* with ACSs from *Escherichia coli*, *Bacillus subtilis*, *Saccharopolyspora erythraea*, and *Mycobacterium smegmatis*. The phylogenic tree was generated using the Phylogeny.fr platform (Dereeper et al., 2008). **(C)** The transcript levels of the four *acs* genes in the original strain A30 grown in TSB liquid medium for 36 and 48 h separately. Fold changes represent the level of expression compared to the expression of the gene *slnwt_2998*. Error bars show standard deviation from three independent experiments.

Based on the KEGG database^2^, there are four putative genes (*slnwt_0620*, *slnwt_6888*, *slnwt_2998*, *slnwt_6934)* encoding AMP-forming acetyl-CoA synthases (EC6.2.1.1, Acs). Generally, there are three conserved domains in AMP-forming acetyl-CoA synthases: Acs encoded by the gene *slnwt_2998* or *slnwt_6934* have the whole three conserved domains, however, Acs translated by *slnwt_0620* or *slnwt_6888* lacks the ASAS_N domain (Figure 2A). In order to identify distinctions among the four isozymes, a phylogenetic tree analysis was performed between the Acs from *S. albus* and other microorganisms, including YtcI of *Bacillus subtilis*, Acs of *E. coli*, *S. erythraea*, and *Mycobacterium smegmatis*. Notably, *slnwt_0620* and *slnwt_6888* had 69.35% identity in amino acid sequence, showing a high homology with AcsA4 of *M. smegmatis*. Acetyl-CoA synthases, encoded by gene *slnwt_2998* and *slnwt_6934*, shared a 39% similarity in amino acid sequence, and they showed a high degree of homology with Acs of *E. coli*, *S. erythraea*, and *M. smegmatis* (Figure 2B). In order to study the transcript level of these four genes (*slnwt_0620*, *slnwt_6888*, *slnwt_2998*, *slnwt_6934*) in *S. albus* at different growth phases, RT-qPCR analysis was conducted when *S. albus* and its mutants were grown in TSB liquid medium for 36 and 48 h, respectively. As shown in Figure 2C, at the early stage of growth phase (36 h), the transcript level of the gene *slnwt_2998* was the highest, followed by *slnwt_6934*, and the transcript level of gene *slnwt_0620* was the lowest. At the end of the growth phase (48 h), the transcript level of *slnwt_2998* was decreased, while transcription of *slnwt_6888* was increased and *slnwt_0620* continued to maintain a very low level. It is assumed that Acs encoded by *slnwt_2998* might play a major catalytic role than Acs encoded by *slnwt_0620* due to its lowest transcript level in *S. albus*.

**FIGURE 3:**
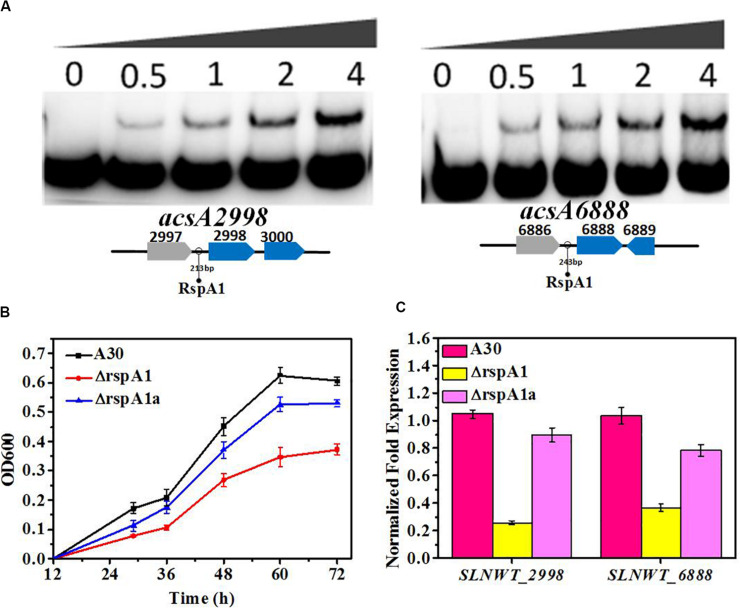
RspA1A2 system directly regulates the transcription of *acsA* in *S. albus*. **(A)** EMSAs of His-RspA1 protein with upstream promoter regions of *slnwt_2998* and *slnwt_6888*. The DNA probe (10 ng) was incubated with a protein concentration gradient (0, 0.5, 1, and 4.0 μg). An excess of poly(d[I-C]) was included in every lane as an internal control to avoid non-specific binding of the protein to the DNA. **(B)** Growth curves of shaking flask fermentation in MM medium complemented with 75 mM glutamate with 0.4% acetate as the sole carbon. OD_600_ was detected to represent the cell density of *S. albus*. **(C)** Transcript level of genes *slnwt_2998* and *slnwt_6888* in A30 rspA1-deletion strain ΔrspA1 and complementary strain ΔrspA1a at 48 h.

To evaluate the impact of *rspA1* mutation on the expression of Acs in *S. albus*, the MM medium with 0.4% acetate as the sole carbon source was used in shaking flask culture. As presented in Figure 3B, the deletion of *rspA1* impaired the cell growth of *S. albus* grown on acetate as carbon source with a 50% decrease in biomass as compared to the original strain A30. However, the growth of complementary mutant ΔrspA1a was restored to some extent when gene *rspA1* was complemented into mutant ΔrspA1. It indicates that the growth defect of mutant ΔrspA1 was due to the absence of *rspA1*. In order to investigate how RspA1 acts on Acs genes, promoter regions of these four genes (−250 to 50 bp from the translation site ATG) encoding Acs were amplified and labeled by biotin to perform electrophoretic mobility shift assays. EMSA results revealed that protein RspA1 could directly bind to the promoter regions of genes *slnw_2998* and *slnwt_6888*, respectively, as DNA probes containing its corresponding promoter region clearly shifted when incubated with the purified recombinant protein RspA1 (Figure 3A). It is suggested that the ACS genes *slnwt_2998* and *slnwt_6888* of *S. albus* were subjected to transcriptional regulation by protein RspA1. However, according to the facts, the promoters of genes *slnwt_0620* and *slnwt_6934* were not bound by the response regulator protein RspA1 (Supplementary Figure S4), and there was a very low transcript level in *S. albus* (Figure 2C). Therefore, it was concluded that the Acs encoded by *slnwt_0620* and *slnwt_6934* could not play a dominant catalytic activity in *S. albus* A30. Meanwhile, the results obtained from qRT-PCR showed that the transcription level of genes *slnwt_2998* and *slnwt_6888* was significantly down-regulated in the Δ*rspA1* mutant as compared to the original strain A30, and this decrease was mostly restored in the complemented strain ΔrspA1a (Figure 3C). To this end, all these findings demonstrated that the protein RspA1 could directly activate the transcription of *acsA* genes *slnwt_2998* and *slnwt_6888* in *S. albus*, followed by Acs translation, which ultimately affects the consumption of carbon source.

### The Transcription of the Citrate Synthase Gene Was Directly Controlled by RspA1

**FIGURE 4:**
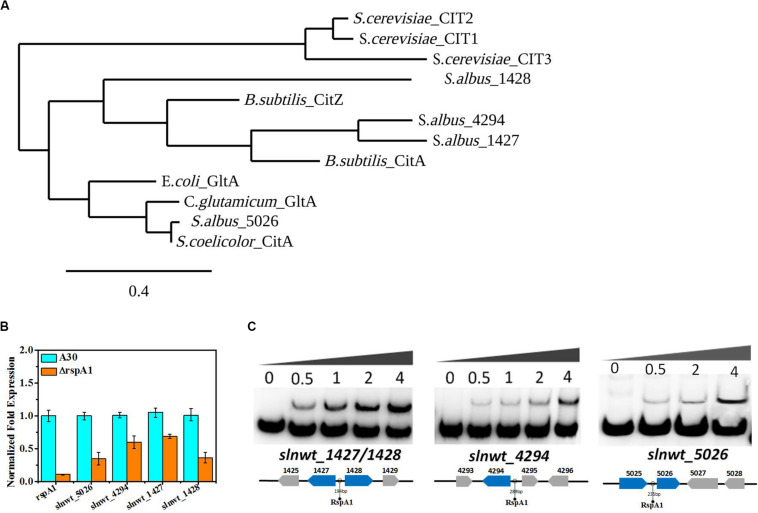
RspA1 directly controls the transcription of citrate synthase. **(A)** Phylogenetic analysis of CSs of *Saccharopolyspora erythraea* with CSs from *Escherichia coli* (GltA,YP_006128080), *Bacillus subtilis* (CitZ,WP_003223555; CitA,WP_003244745), *Corynebacterium glutamicum* (GltA, WP_015439438), *Saccharomyces cerevisiae* (CIT1, EDN62808; CIT2, EDN62126; CIT3, EDN61138), and *Streptomyces coelicolor* (CitA, CAB66275). The phylogenic tree was generated using the Phylogeny.fr platform (Dereeper et al., 2008). **(B)** The transcript levels of the four CSs genes in the original strain A30 and mutant ΔrspA1 grown in a fermentation medium at 36 h. Error bars show standard deviation from three independent experiments. **(C)** EMSAs of His-RspA1 protein with upstream promoter regions of *slnwt_1427*, *slnwt_1428*, *slnwt_4294*, and *slnwt_5026*. The DNA probe (10 ng) was incubated with a protein concentration gradient (0, 0.5, 1, and 4.0 μg). An excess of poly(d[I-C]) was included in every lane as an internal control to avoid non-specific binding of the protein to the DNA.

Citrate synthase (CS, EC 2.3.3.1) catalyzes the initial reaction of the TCA cycle and influences the glucose consumption rate in bacteria. *S. albus* harbors four putative genes encoding citrate synthase (*slnwt_1427*, *slnwt_1428*, *slnwt_4294*, *slnwt_5026*) (KEGG database)^2^. The gap between *slnwt_1427* and *slnwt_1428* is 194 bp, and they have an opposite translation direction, indicating that these two genes may share the same promoter. In order to predict the functions of these four genes, a phylogenetic tree was created between CS from *S. albus* and other microorganisms (*S. erythraea*, *E. coli*, *C. glutamicum*, *B. subtilis*, and *S. coelicolor*). The results showed that citrate synthases encoded by *slnwt_1427* and *slnwt_4294* had 42% identity in amino acid sequence and exhibited partial similarity with the CitA of *B. subtilis* (25% for *slnwt_1427* and 25.3% for *slnwt_4294*). Interestingly, citrate synthases encoded by *slnwt_5026* showed 90.85% similarity with the CitA of *S. coelicolor*. However, the citrate synthase encoded by *slnwt_1428* in *S. albus* is distinctive from traditional citrate synthase from other bacterial species (Figure 4A).

Besides that, the TCA cycle also plays a crucial role in the utilization of carbon sources. Therefore, it is essential to check the transcript level of genes encoding the key enzyme citrate synthase in the TCA cycle. The results showed that the transcript level of genes (*slnwt_1427*, *slnwt_1428*, *slnwt_4294*, *slnwt_5026*) had a significant decrease in mutant ΔrspA1 as compared with the original strain (Figure 4B). As citrate synthase is located at the entry point of the TCA pathway, its lower transcription level indicated that the flux of the TCA cycle was reduced to some extent, leading to less-consumed glucose in *rspA1* gene-deficient mutant ΔrspA1. Unexpectedly, electrophoretic mobility shift assay results also revealed that protein RspA1 could directly bind to the promoter regions of the genes *slnwt_1427*, *slnwt_1428*, *slnwt_4294*, and *slnwt_5026*, respectively (Figure 4C). Conclusively, these findings demonstrated that the transcription of citrate synthase was directly activated by protein RspA1, and the precise regulation of the conversion of acetyl-CoA to TCA cycle was controlled by RspA1 in *S. albus.*

### The PEP–Pyruvate–Oxaloacetate Node Was Regulated by Protein RspA1

The PEP-pyruvate-oxaloacetate node was located in the metabolic link between glycolysis/gluconeogenesis and the TCA cycle which, acts as a highly central control point for glucose consumption and glucose-generating metabolism (Sauer and Eikmanns, 2005). In this study, we found that RspA1 could regulate the transcription of the gene *pdh* encoding PDH in the glycolysis pathway and CS gene transcription at the TCA cycle. Therefore, it can be hypothesized that the glucose consumption rate was strongly regulated by RspA1 via the PEP–pyruvate–oxaloacetate node in *S. albus*.

**FIGURE 5:**
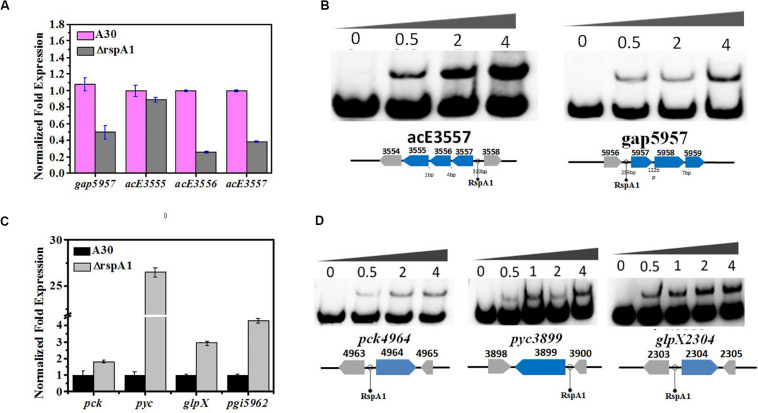
RspA1A2 system regulates the PEP–pyruvate–oxaloacetate node in *S. albus*. **(A)** Transcription level of genes *slnwt_5957*, *slnwt_3555*–*3557* in A30 and *rspA1*-deletion strain (ΔrspA1). **(B)** EMSAs of His-RspA1 protein with upstream promoter regions of *slnwt_3557* and *slnwt_5957*. The DNA probe (10 ng) was incubated with a protein concentration gradient (0, 0.5, and 4.0 μg). **(C)** Transcript level of genes *pck*, *pyc*, *glpX*, and *pgi* in A30 and *rspA1*-deletion strain. **(D)** EMSAs of His-RspA1 protein with upstream promoter regions of *pck, pyc*, and *glpX*. The DNA probe (10 ng) was incubated with a protein concentration gradient (0, 0.5, 1.0, 2.0, and 4.0 μg). An excess of poly(d[I-C]) was included in every lane as an internal control to avoid non-specific binding of the protein to the DNA.

Under glycolytic conditions, the final products of glycolysis PEP and pyruvate entered the TCA cycle via acetyl-CoA (oxidative pyruvate decarboxylation and fueling of the cycle). So, we firstly checked the transcript level of key genes in the flux of pyruvate transformation to acetyl-CoA by qRT-PCR analysis, and these are the genes that encode PDH (*acE*, *slnwt* _3555–*slnwt* _3557). The gap between genes *slnwt* _3555–*slnwt* _3557 was 1 and 4 bp, indicating that these three genes were co-transcribed and encoded PDH to catalyze pyruvate transformation into acetyl-CoA. The results obtained from qRT-PCR analysis revealed that the transcript level of genes encoding PDH (*slnwt*_3556–*slnwt*_3557) was down-regulated in mutant ΔrspA1 as compared to the original strain A30 (Figure 5A), which means that the conversion of pyruvate into acetyl-CoA was decreased on account of the *rspA1* defect. Interestingly, it was also found that the transcript level of genes encoding glyceraldehyde-3-phosphate dehydrogenase (G3P) (*gap*, *slnwt_*5957) was declined in mutant ΔrspA1 (Figure 5A). Moreover, the expression of genes *pfkA* (*slnwt_1861*, *slnwt_7260*) and *pyk* (*slnwt_1865*, *slnwt_5886*) showed no corresponding changes in mutant ΔrspA1 (Supplementary Figure S5). Then, we further explored whether the protein RspA1 could directly bind to the promoter of these genes. Therefore, the putative promoter regions (+50 bp to −250 bp from the translation site ATG) of the genes *slnwt_*5957 and *slnwt*_3555–*slnwt*_3557 were amplified and labeled by biotin. As shown in Figure 5B, the protein RspA1 could directly bind to the promoter regions of genes *slnwt_*5957 and *slnwt*_3555–*slnwt*_3557. Hence, these EMSA results provided us a solid evidence to support the fact that RspA1 can promote the expression of the gene *pdh* and directly control the flux of pyruvate into acetyl-CoA.

As *rspA1* defect decreased the flux of pyruvate transformation into acetyl-CoA, to identify a potential adaptive and anaplerotic pathway by which mutant ΔrspA1 maintains the balance of pyruvate, transcription analysis for the flux of gluconeogenesis was carried out. The expression of three key genes in the gluconeogenesis pathway was checked, which were *pyc*, *pck, glpX*, and *pgi* (pyc, *slnwt_3899*; *pck*, *slnwt_4964*; *glpX*, *slnwt_2304*; *pgi*, *slnwt_5962*). Interestingly, the expression of genes *pyc*, *pck, glpX*, and *pgi* was significantly up-regulated in mutant ΔrspA1 (Figure 5C). Besides that, the results obtained from EMSAs showed that RspA1 could directly bind the promoter regions of *pyc*, *pck*, and *glpX* (Figure 5D). Consequently, these findings revealed the RspA1-dependent regulation of the PEP-pyruvate-oxaloacetate node. Specifically, on the one hand, *rspA1* defect directly decreased the flux of pyruvate transformation into acetyl-CoA; on the other hand, *rspA1* defect increased the flux of gluconeogenesis to produce glycogen. This regulation strategy can ensure appropriate distribution between anaplerotic/gluconeogenic (PEP synthase) and catabolic (pyruvate dehydrogenase complex, PDH) flux when both enzymes compete for the pyruvate substrate in *S. albus*.

### Differentially Expressed Genes (DEGs) in Primary Metabolism Pathway Between Mutant ΔrspA1 and the Initial Strain A30

**FIGURE 6:**
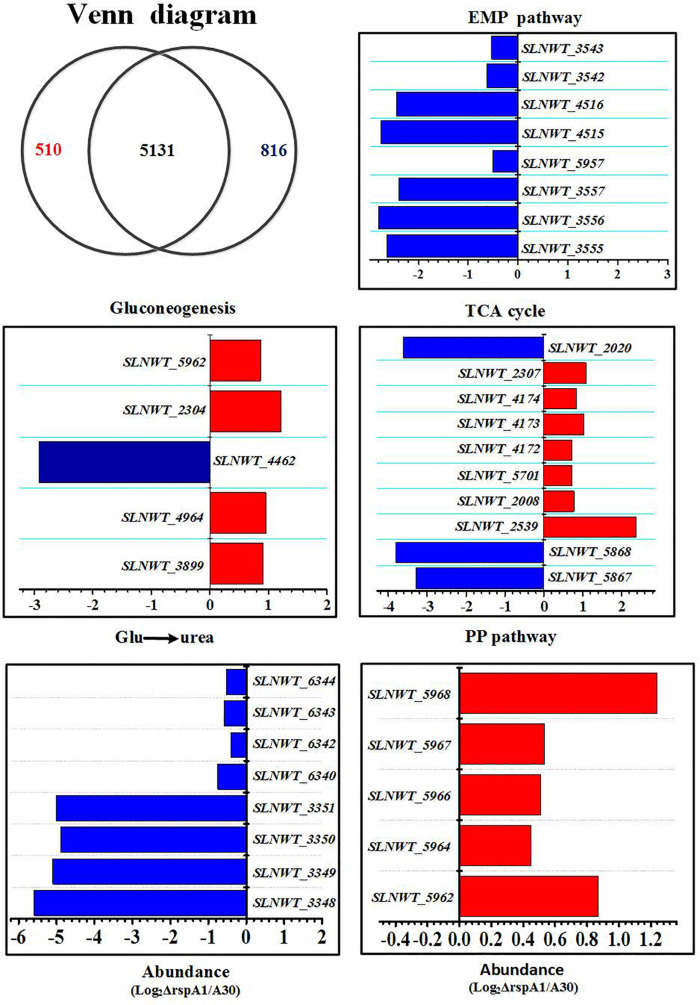
Transcriptomic analysis of the ΔrspA1 mutant and the original strain A30. Mutant ΔrspA1 vs. the original strain A30 at 30 h on fermentation cultures complemented with 75 mM glutamate in shaking flask culture. Venn diagram shows transcripts with significant up-regulation (*p* < 0.03) in the original strain A30 (816 transcripts) or mutant ΔrspA1 (510 transcripts), and transcripts without significant variations (5131 transcripts). Histograms show the abundance of the transcripts with significant variations (*p* < 0.03) discussed in the text. Abundance values (average from two biological replicates) are shown. For details of genes expression, please see Supplementary Table S2.

In order to understand the global regulation of TCS RspA1/A2 on carbon source metabolism at transcriptional level, the transcriptomic datasets between mutant ΔrspA1 and the original strain A30 were compared at the early growth phase (30 h) in a fermentation medium complemented with 75 mM glutamate in shaking flask culture. As a result, 1326 genes showed altered expression in mutant ΔrspA1 as compared with the original strain A30 (Figure 6). As expected, the *rspA1* transcript was absent in the ΔrspA1 mutant (Supplementary Table S2). Out of 1326 transcripts, 510, which are 38% of the total, were up-regulated in the ΔrspA1 mutant as compared to the initial strain A30, whereas 816 out of 1326 transcripts exhibited up-regulation and 5131 transcripts did not show significant variations (Figure 6 and Supplementary Figure S6).

**FIGURE 7:**
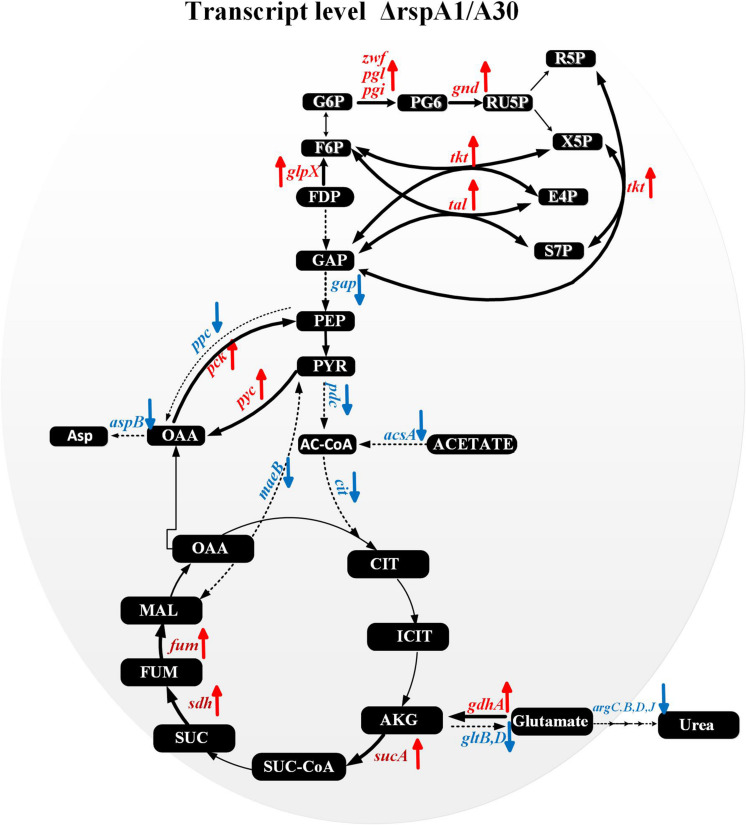
Transcriptomic data analysis of differentially expressed genes (DEGs) in the mutant strain ΔrspA1 compared with the initial strain A30 in primary metabolism. → Up-regulated in mutant ΔrspA1; ---→ Down-regulated in mutant ΔrspA1. For detail genes expression, please see Supplementary Table S2.

Several key genes involved in primary metabolism were differentially expressed in mutant ΔrspA1 as compared to the initial strain A30 (Figure 6). Herein, our attention was focused on DEGs located in the EMP pathway, the TCA cycle, gluconeogenesis, and the pentose phosphate pathway (PPP pathway). The expression of genes located in the EMP pathway was strikingly down-regulated in mutant ΔrspA1, including genes encoding PDH (*slnwt* _3555–*slnwt* _3557, *slnwt_4115*–*slnwt_4116*, *slnwt_3542*–*slnwt_3543*), and the gene encoding glyceraldehyde-3-phosphate dehydrogenase (*gap*, *slnwt_5957)* (Figures 6, 7). The transcript level of genes encoding the key enzymes located in the gluconeogenesis pathway exhibited variations in the mutant: the genes *pyc*, *pck*, and *glpX* involved in flux of pyruvate transformation into glucose were up-regulated, and the expression of the gene (*ppc*, *slnwt_4462)* encoding phosphoenolpyruvate carboxylase to catalyze PEP transformation into oxaloacetate (OAA) was down-regulated in mutant ΔrspA1 (Figures 6, 7). These findings were consistent with previous results obtained from qRT-PCR (Figure 5). Moreover, these findings demonstrated that the *rspA1* defect markedly enhanced pyruvate-driven gluconeogenesis and decreased the flux of pyruvate into acetyl-CoA. In addition, the flux of PPP pathway was enhanced due to the increased flux of the gluconeogenesis pathway which directly provided precursors for the PPP pathway (Figures 6, 7). The expression of the genes (*sdh*, *slnwt_4172*–*slnwt_4174*; *gdhA*, *slnwt_2539*; *sucA*, *slnwt_2008* and *slnwt_5701*; *fum*, *slnwt_2307*, and *slnwt_2312*) involved in the flux of glutamate transformation into oxaloacetate through the TCA cycle was up-regulated; the genes *meaB*, *slnwt_2020* and *aspB*, *slnwt_6615* were significantly down-regulated in mutant ΔrspA1. In contrast, the flux of glutamate converting into a urea cycle was decreased, whereas the genes *argB*: *slnwt_3350*, *slnwt_6343*; *argC*: *slnwt_3348*, *slnwt_6340*; *argD*: *slnwt_3351*, *slnwt_6344*; *argJ*: *slnwt_3349*, *slnwt_6342* were significantly down-regulated in mutant ΔrspA1 (Figures 6, 7). Moreover, the results suggested that the *rspA1* defect increased the flux of glutamate transformation into oxaloacetate through the TCA cycle but decreased the flux of glutamate transformation into a urea cycle. In summary, the flux of glutamate transformation into oxaloacetate is an alternative way to compensate the decreasing flux of pyruvate into the TCA cycle when mutant ΔrspA1 is cultured with the glutamate-rich medium.

## Discussion

In the current study, the mutants with deletion and complementation of the *rspA1* gene encoding the response regulator RspA1 of TCS RspA1/A2 have been genetically generated. RspA1-binding sites are identified in the promoters of key genes located in the glycolysis pathway, the TCA cycle, and gluconeogenesis using an electrophoretic mobility shift assay. Based on EMSA results, qRT-PCR, and the transcriptomic data of mutant ΔrspA1 and the parental strain A30, we unveiled the effects of the TCS RspA1/A2 on the central carbon metabolic pathways in *S. albus* when cultivated with glutamate as the sole nitrogen source.

### The Regulation of TCS RspA1/A2 on Glucose Consumption Rate Is Conditionally Required

Carbon source regulation of antibiotic production has been widely studied in the past few decades. Especially, glucose as the preferred carbon source has been deemed to interfere with antibiotic synthesis in *Streptomyces*. Only the low concentration of glucose in the culture medium can eliminate the repression of antibiotic biosynthesis, and its regulatory mechanism of carbon catabolite repression (CCR) has already been elucidated in previous studies (Deutscher, 2008; Görke and Stülke, 2008). However, the regulation of carbon metabolism for cell growth in *Streptomyces* has been unclear when cultivated under a specific condition. In this work, we found that the cell growth of *rspA1* knocked-out mutant ΔrspA1 was hampered with less biomass as compared to the initial strain A30 when cultured in the MM medium complemented with 75 mM glutamate. However, this effect seems to be conditionally required and only worked under specific environmental conditions, which is similar to the most two-component systems studied in *Streptomyces* (Apel et al., 2007; Shu et al., 2009). When the sodium glutamate was replaced with an identical concentration of phenylalanine (Phe), histidine (His), and aspartate (Asp), respectively, the initial strain A30 and the *rspA1* knocked-out strain did not show significant growth differences, indicating that the regulator RspA1 responded to specific amino acids. In most bacteria, glutamate could be transformed into α-ketoglutarate (α-KG) under the catalysis of glutamate dehydrogenase (GdhA). Therefore, glutamate cannot only be used as a nitrogen source, but it also has a key node for the conversion of nitrogen and carbon sources in the microorganism. Moreover, with the sufficient amount of glutamate as a nitrogen source in *S. albus*, the protein RspA1 can respond to keep nitrogen and carbon sources at a delicate balance. In addition to the impairment of the biomass of *S. albus* in mutant ΔrspA1, it was found that the glucose consumption rate of mutant ΔrspA1 was significantly reduced as compared to the initial strain A30. Glycolysis, gluconeogenesis, and the TCA cycle are involved in glucose metabolism. Therefore, changes in the rate of glucose consumption are related to changes in metabolic fluxes through these central carbon metabolic pathways.

### Two-Component-System RspA1/A2-Dependent Regulation of the PEP–Pyruvate–Oxaloacetate Node Affects the Intracellular Acetyl-CoA Pool in *S. albus*

**FIGURE 8:**
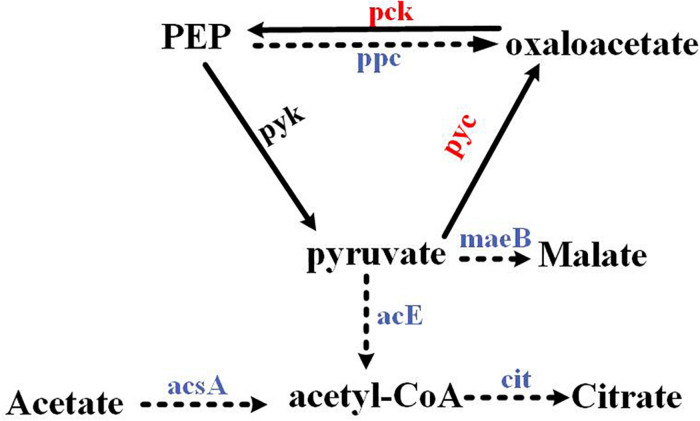
The PEP–pyruvate–oxaloacetate node was directly regulated by the response regulator RspA1. → Up-regulated in the ΔrspA1 mutant; ---→ Down-regulated in the ΔrspA1 mutant.

In organisms, pyruvate, oxaloacetate, and phosphoenolpyruvate (PEP) could be converted into each other. Herein, we demonstrated that the PEP–pyruvate–oxaloacetate node was directly regulated by the response regulator RspA1 (Figure 8), and the deletion of gene *rspA1* affected the intracellular acetyl-CoA pool in mutant ΔrspA1, which led to less intermediate formation for biomass accumulation. Firstly, the response regulator RspA1 directly regulated the supply of intracellular acetyl-CoA. This study has demonstrated that the protein RspA1 could directly interact with the promoter regions of genes *slnwt_0620*, *slnwt_6888* encoding the acetyl-CoA synthase (Acs) and promoted their expression in *S. albus*. Acetyl-CoA synthase catalyzed the synthesis of acetyl-CoA with acetate as the substrate, whereas the *rspA1* defect inhibited the expression of acetyl-CoA synthase and restrained the flux of acetate converted to acetyl-CoA (Figures 3, 8). Meanwhile, the expression of genes encoding the PDH (acE, *slnwt* _3555–*slnwt* _3557) was directly inhibited due to the absence of protein RspA1. These three genes were co-transcribed and encoded an enzyme complex PDH to catalyze the conversion of pyruvate into acetyl-coA (Figures 5, 8). The synergetic regulation of enzymes including the PDH and acetyl-CoA synthetase Acs could lead to the decreased supply of intracellular acetyl-CoA. Secondly, the protein RspA1 regulated the disposition of intracellular acetyl-CoA. The expression of genes encoding citrate synthase (*slnwt_1427*, *slnwt_1428*, *slnwt_4294*, *slnwt_5026*) was decreased by the *rspA1* defect, thus affecting the acetyl-CoA entry into the TCA cycle. Consequently, this combined regulation of the PEP–pyruvate–oxaloacetate node could lead to a decrease in intracellular acetyl-CoA. As we know, acetyl-CoA is a crucial precursor for many secondary metabolites, and its reduction could limit the synthesis of secondary metabolites as well as antibiotics. This finding also clarifies the results of our previous study, in which the production of salinomycin decreased in the ΔrspA1 mutant (unpublished). Additionally, protein RspA1 could directly regulate the key genes in gluconeogenesis. At last, the results of qRT-PCR analysis showed that the expression of the genes *pyc*, *pck* was strikingly up-regulated in the ΔrspA1 mutant, thus enhancing the flux of pyruvate converting into acetoacetate and phosphoenolpyruvate (Figures 5, 8).

### Pyruvate and Glutamate Transformations Enable TCS RspA1/A2-Dependent Regulation of Primary Metabolism in *Streptomyces albus*

**FIGURE 9:**
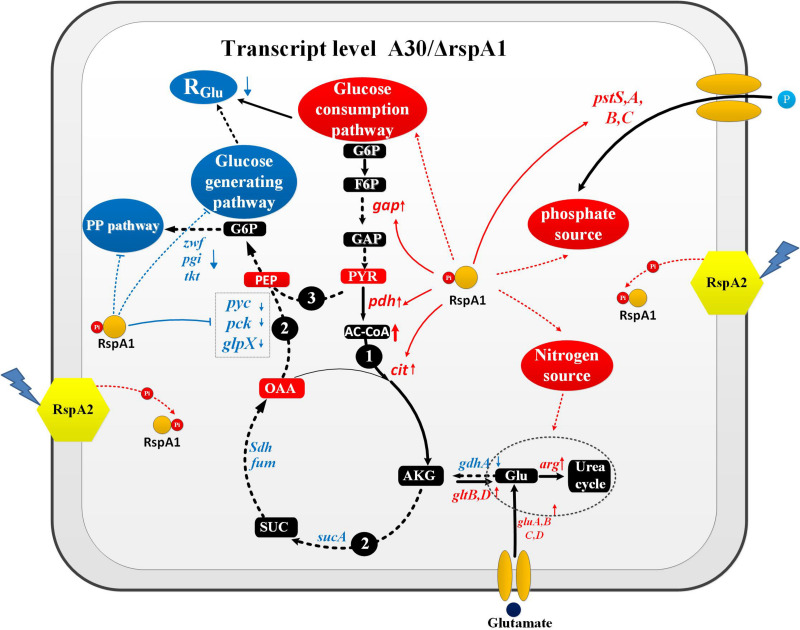
Pyruvate and glutamate transformations enable TCS RspA1/A2-dependent regulation of glucose metabolism in *Streptomyces albus*. Pathway 1 represents the flux of pyruvate converting to TCA cycle; Pathway 2 represents the flux of glutamate transformation to glucose through TCA cycle; Pathway 3 represents gluconeogenesis pathway. → Up-regulated in the original strain A30; ---→ Down-regulated in the original strain A30; → Positive control; —| Negative control.

A new transcriptional regulatory network of TCS RspA1/A2 on primary metabolism across central carbon metabolic pathways including the glycolysis/gluconeogenesis pathway, TCA cycle, and PPP pathway was proposed. Furthermore, its transcriptional regulatory mechanism can be speculated as follows:

The protein RspA1 increased the glucose consumption rate (R_Glu_) by enhancing the pyruvate-driven glucose consumption pathway and decreasing the glutamate-driven gluconeogenesis pathway in the original strain A30 as compared with mutant ΔrspA1 (Figure 9, pathway 1–3). We firstly demonstrated that RspA1 could bind the promoter regions of the genes *gap*, *pdh*, and *cit* and directly promote their expression (Figure 6B). PDH and citrate synthase were located at the entry point of the TCA pathways, and their increasing expression indicated that the flux of pyruvate to the TCA cycle was strengthened. The central carbon metabolism through the EMP and TCA pathways played a dominant role in the metabolism of glucose, and their strengthened flux would result in an increase in glucose consumption (Figure 9, pathway 1). Meanwhile, the flux of the glucose-generating pathway was decreased. Gluconeogenesis is the main glucose-generating pathway in bacteria (Landau et al., 1996), and transcriptome analysis revealed that the key genes (*pyc*, *pck*, and *glpX*) in the gluconeogenesis pathway (pyruvate to glucose) and genes *sdh*, *gdhA*, *sucA*, *fum* in the flux of glutamate transformation to oxaloacetate through TCA cycle were significantly down-regulated in the original strain A30 (Figure 9,pathways 2 and 3). Furthermore, this suggested that the protein RspA1 enhanced the gluconeogenesis flux with pyruvate and glutamate as substrates when cultivated in a glutamate-rich medium. In short, pyruvate and glutamate transformations supported the robust TCS RspA1/A2-dependent regulation of glucose metabolism, resulting in an increased flux of glucose consumption and a decreased flux of glucose-generating pathway in the original strain A30 as compared with mutant ΔrspA1. Simultaneously, this kind of regulation may account for the lower glucose consumption rate in mutant ΔrspA1 as compared to the initial strain A30 (Figure 1D). In mammals like mice, glutaminolysis and pyruvate transformation support MPC-independent gluconeogenesis when the flux of pyruvate entry into the TCA cycle was decreased by the deletion of the gene *Mpc1.* In this study, we discovered a similar regulation mechanism on glucose metabolism in *Streptomyces* (Gray et al., 2015).

On the other hand, the protein rspA1 regulated the interaction between glucose metabolism, nitrogen metabolism, and phosphate metabolism in order to keep them balanced. At the early stage of the growth phase (30 h), glucose was broken down into pyruvate, and the flux of pyruvate entry into the TCA cycle was enhanced (Figure 9). In contrast, the genes *pyc*, *pck*, and *glpX* in the gluconeogenesis pathway were inhibited by the protein RspA1 in the original strain A30, indicating that glucose catabolism was more active than glucose anabolism (Figure 9). Additionally, with sufficient glutamate (75 mM) as the nitrogen source, the expression of genes *gluA-D* encoding the glutamate transporters (ABC transporters) were prominently up-regulated to strengthen the uptake of glutamate. Interestingly, we found that the flux of glutamate converting to a urea cycle was enhanced due to the expression of genes *argB*, *C*, *D*, *J*, which shows an obvious up-regulation in the original strain A30 (Supplementary Figure S7). Therefore, it is concluded that the leftover glutamate was more transferred to a urea cycle other than carbon metabolism due to active glucose catabolism. As we know, a urea cycle is not the only way to biosynthesize some amino acids like arginine and citrulline but also provides cells with precursors for DNA replication and other intermediates. In addition, we demonstrated that protein RspA1 had a direct regulation of the uptake of phosphate sources. The EMSA results show that protein RspA1 could directly bind the promoter region of the gene *pstS* (*slnwt_4043*), which encoded an ABC transporter response for the uptake of phosphate sources (Supplementary Figure S7). The genes *pstA*, *pstB*, and *pstC* were located at the upstream of gene *pstS* and worked together with p*stS* in response to the uptake of phosphate sources; meanwhile, transcriptome analysis revealed that the expression of the genes *pstA*, *B*, *C*, *S* was strikingly increased in the original strain A30 (Supplementary Figure S7). Moreover, these findings suggested that TCS RspA1 promoted the uptake of the phosphate source. In summary, RspA1 promoted glucose catabolism, the uptake of phosphate sources, and the flux of glutamate into a urea cycle but decreased glucose anabolism. In addition, this combined regulation of primary metabolism may explain the fact that the cell growth of *rspA1* knocked-out mutant ΔrspA1 was hampered with less biomass accumulation as compared to the initial strain A30.

In this study, our main aim focus was on the momentous regulation of RspA1/A2 on primary metabolism (cell growth and glucose metabolism) in *Streptomyces albus*, which may in turn affect secondary metabolism. In fact, the secondary metabolites are synthesized via secondary metabolism, which shows a coupled relationship with primary metabolism. Moreover, the metabolic pathways of primary metabolism are not only providing essential support for cellular growth but also supplying precursors and energy for secondary metabolism. For example, acetyl-CoA, Malonyl-CoA(M-CoAs), and methylmalonyl-CoA(MM-CoAs), which are the vital intermediate metabolites in the TCA cycle, are considered as the main precursors of salinomycin biosynthesis (Lu et al., 2016). So, the regulation of RspA1/A2 on the flux of the TCA cycle will surely alter the intracellular pools of these precursors. Besides that, the regulation of the PEP–pyruvate–oxaloacetate node by RspA1/A2 was demonstrated, which is very common in bacteria (Sauer and Eikmanns, 2005). One interesting feature of the PEP–pyruvate–oxaloacetate node is the fact that it also provides the precursors for antibiotic biosynthesis which could open another new door/way toward improving antibiotic production (Cruz, 2012). Moreover, it is highly valuable to discover the effect of the PEP–pyruvate–oxaloacetate node on the secondary metabolism in *Streptomyces.* However, this needs to be explored, and further research studies are required in future works.

## Data Availability Statement

Publicly available datasets were analyzed in this study. This data can be found here: Raw data are available via the Gene Expression Omnibus database (accession GSE143602).

## Author Contributions

MG and KZ conceived the project, designed the experiments, and analyzed the results. KZ wrote the manuscript with the help of AM, MA, MG, YZ, and JC. KZ performed the experiments, supported by JY, YH, and ZC. All authors saw and approved the manuscript and contributed significantly to the work.

## Conflict of Interest

ZC was employed by Zhejiang Biok Biology Co., Ltd. The remaining authors declare that the research was conducted in the absence of any commercial or financial relationships that could be construed as a potential conflict of interest.

## Footnotes

1 https://www.ncbi.nlm.nih.gov/nuccore/NZ_CP010519.1

2 https://www.kegg.jp/
