# Supplementary material for: Two-Component-System RspA1/A2-Dependent Regulation on Primary Metabolism in Streptomyces albus A30 Cultivated With Glutamate as the Sole Nitrogen Source
Source: Front Microbiol. 2020 Jul 31;11:1658. doi: 10.3389/fmicb.2020.01658 (PMC7411085; doi:10.3389/fmicb.2020.01658)
Supplement: Supplementary file 1 [file Data_Sheet_1.docx]

**Figure S1 .**validation of RNA-seq Δ*rspA1* mutant results using RT-qPCR. correlation between RNA-seq and RT-qPCR abundance values (both relative to the initial strain A30 ) for eight genes.


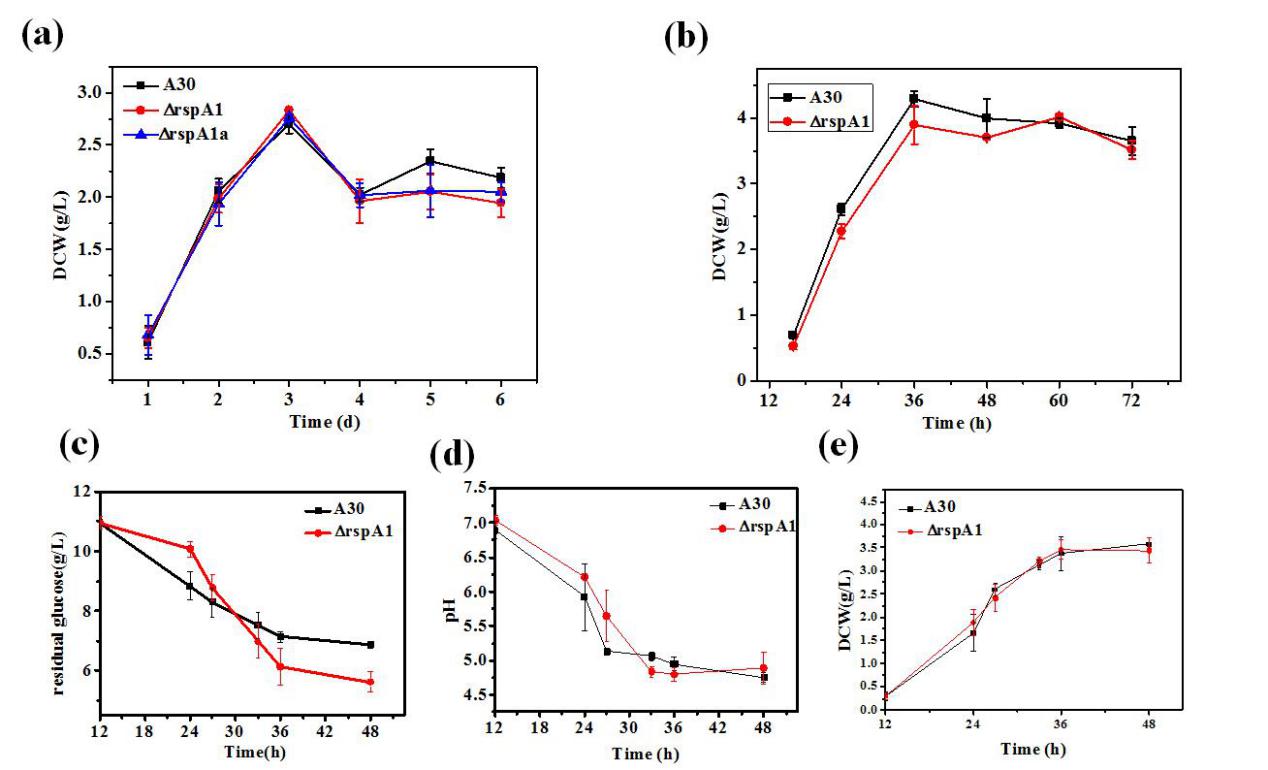


**Figure S2.** Mutation of rspA1 affects biomass accumulation and glucose consumption rate in *Streptomyces albus*.( A-B).Growth differences between the mutant ΔrspA1, ΔrspA1a and the initial strain A30 when cultured inISP4 medium (A)or TSB medium(B). (C-E) Residual sugar, pH and dry cell weight(DCW) of the mutant ΔrspA1, ΔrspA1a and the initial strain A30 when cultured in fermentation medium complemented with 75mM sodium glutamate.

**Figure S3.** Growth differences between mutant (ΔrspA1) and the initial strain A30 when cultured in MM solid plate complemented with various amino acid.

**Figure S4.** EMSAs of His-RspA1 protein with upstream promoter regions of *slnwt_0620* and *slnwt_6934*. The DNA probe (10 ng) was incubated with a protein concentration gradient (0, 0.5, 1 and 4.0 ug).


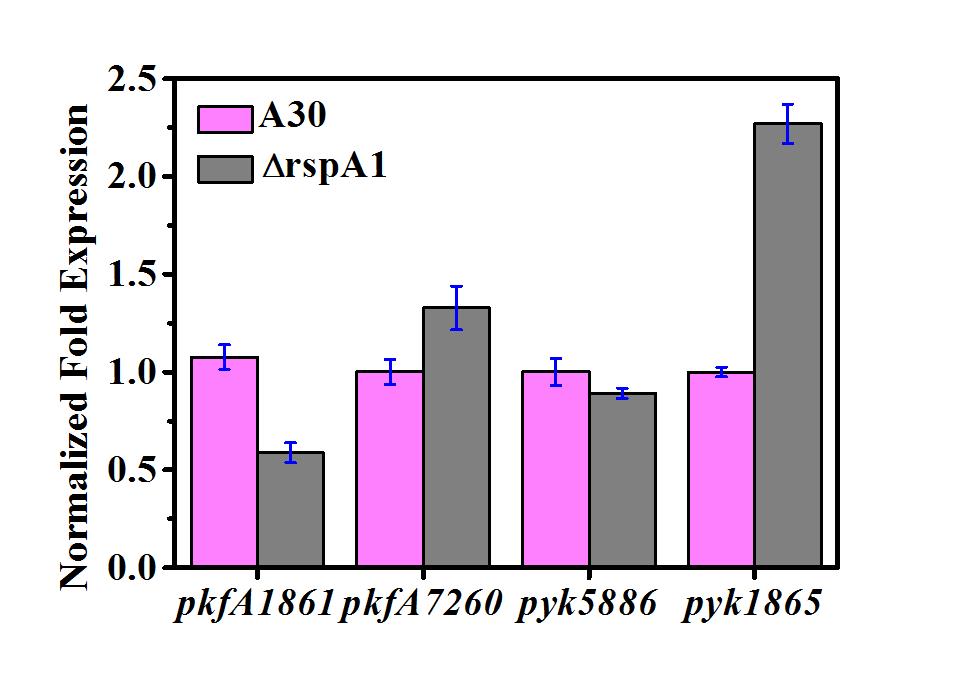


**Figure S5.** Transcript level of genes *pkfA* and *pyk* in A30 and *rspA1*-deletion strain.

**Figure S6 .**The heat map (A)and Volcanoplot(B) of diﬀerentially expressed genes in ΔrspA1 mutant and the initial strain A30.

**
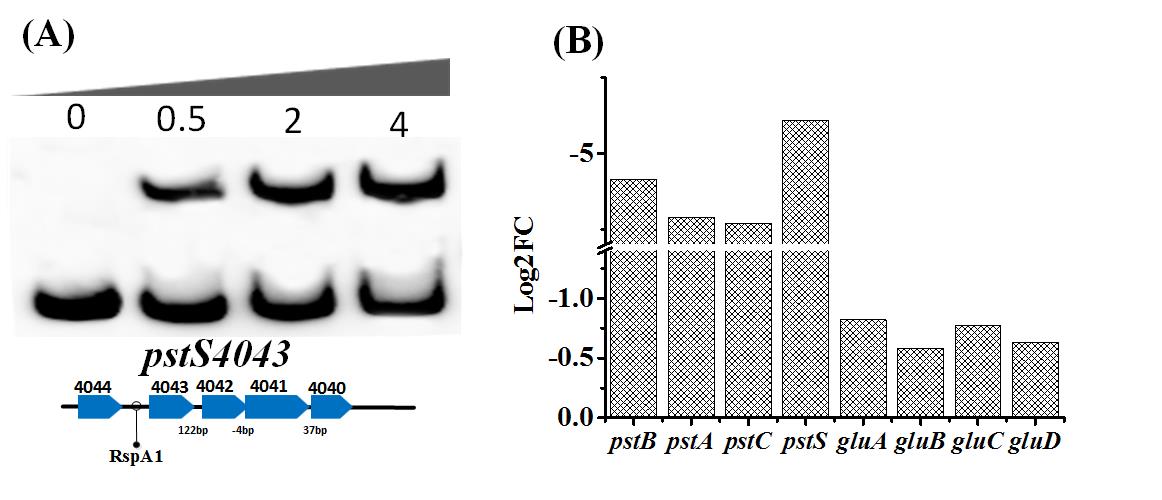
**

**Figure S7.** RspA1 effect the uptake of glutamate and phosphate source **(A)**EMSAs of His-RspA1 protein with upstream promoter regions of *slnwt_4043* a. The DNA probe (10 ng) was incubated with a protein concentration gradient (0, 0.5, 1 and 4.0 ug).(B) transcript level of gene *pstS,A,B,C* and *gluA-B.* For detail genes expression, please see Table S2.

**Supplementary material**

**Tables**

**Table S1.primers used in the study**

| name | sequences（5’-3’） |
| --- | --- |
| Primers for disruption |  |
| pJTU-*rspA1*-up-F | CTCGCCAGTCGATTGGCTGA CGCTGGACATTCTGTCCCATG |
| pJTU-*rspA1*-up-R | TCGACGGTATCGATAAGCTT CTCGCCGTGTTCAAGACCTAC |
| pJTU-*rspA1*-dn-F | CTCGCCAGTCGATTGGCTGA AGGACGAGGTGCTCAGCTTC |
| pJTU-*rspA1*-dn-R | TCGACGGTATCGATAAGCTT CGGACAAGAAGCTCACCCTC |
| Primers for complementation |  |
| *rspA1*-F-*Nde*I | GGTTGGTAGGATCCACAT GTGCCTTCCCTGTTGCTGATC |
| *rspA1*-R-*Eco*RI | CTATGACATGATTACGAATTC  TCATGAGGGAGTGTCCAGCC |
| RT-qPCR primers |  |
| acs0620-RT-F | TTCGAGCTGGAGAGCGCACT |
| acs0620-RT-R | GTGCGCGAAGATCTCCTTCG |
| acs6934-RT-F | GATCAACAACGCCCTGCTGC |
| acs6934-RT-R | CTACACCTTCGGCCTCGAAG |
| glt1427-RT-F | ATCTCGACGATCGACCACGG |
| glt1427-RT-R | ATCCAGGGGTCGATCCGGTC |
| glt4294-RT-F | TGATGGCGCTCTCCTACGTC |
| glt4294-RT-R | GAGGTCCAGTAGGCGTCCAC |
| glt5026-RT-F | ACGAGATCACCCAGCACACC |
| glt5026-RT-R | TCGAGAGATGCCGCTGCTTC |
| glt1428-RT-F | TGCTCGACGAGGTGCTCCAG |
| glt1428-RT-R | CCTCTTCGAGCAGCCCGAAC |
| acE3557-RT-F | ACTTCCACGAGGCGCTGAAC |
| acE3557-RT-R | ACGTCGTTGCCGTCGACCAG |
| pfkA1861-RTF | ACAACGACATCTCCGCCACC |
| pfkA1861-RT-R | TCGACGACCATCACGCGCTG |
| pfkA5764-RT-F | CCTCGCGAAGAACGAGGTGG |
| pfkA5764-RT-R | GTGTAGTCGGTGGCGGAGAG |
| pfkA7260-RT-F | ACAACGACATCGCCGCCACC |
| pfkA7260-RT-R | TCGACGATCAGGACCCGCTG |
| gap5957-RT-F | GTCCTCGACGAGAACTTCGG |
| gap5957-RT-R | CGTCCAGCTTGCCCTTGAGC |
| pgi5962-RT-F | CTACCTCTCGCGGTTCACCG |
| pgi5962-RT-R | TGCCCTGGTGGATCAACTGG |
| pyk1865-RT-F | ACGCCGACGACGTCAAGGAC |
| pyk1865-RT-R | AGGCCATCACCACGGACTCC |
| pyk5886-RT-F | ACGGCGACCACATCCTCATC |
| pyk5886-RT-R | AGGTTGAGGCCCTTGTGGTC |
| ppc4964-RT-F | ACCGCGAGATCTGGTCCTTC |
| ppc4964-RT-R | AGATCGTCGGCTCCAGCATC |
| pyc4964-RT-F | CATCTGCCTCCAGATGCTGC |
| pyc4964-RT-R | TGCGGAAGATGTCGATGCCG |
| glpX2304-RT-F | AGGGCATGTCCAACGCCATC |
| glpX2304-RT-R | GGTGGAGTTCTTCGCCTTGG |
| aceA6749-RT-F | GATCAACAACGCCCTGCTGC |
| aceA6749-RT-R | CGATCATGGCCTTGGTCAGC |
| EMSA primers |  |
| EMglt1427-F | AGCCAGTGGCGATAAG GTCATGGCGTCACCGTATCC |
| EMglt1427-R | AGCCAGTGGCGATAAG TGCTCTGGCCGAGGAGGAAG |
| EMglt4294-F | AGCCAGTGGCGATAAG ATCCTGCCGCATCCGGCACC |
| EMglt4294-R | AGCCAGTGGCGATAAG CGGGTACGAAGTCGGACATC |
| EMglt5026-F | AGCCAGTGGCGATAAG AGGTCACACTGCTCTTCCGG |
| EMglt5026-R | AGCCAGTGGCGATAAG TCTGGGTGTTGTCGCTCACG |
| EMacs0620-F | AGCCAGTGGCGATAAG CGGCTGATGTCCCGGATGTG |
| EMacs0620-R | AGCCAGTGGCGATAAG GTGGGCGTCATCAGTCCTCC |
| EMacs2998-F | AGCCAGTGGCGATAAG AGAGACGGAGAAGGCGAGGG |
| EMacs2998-R | AGCCAGTGGCGATAAG CGATCGACGGACGCTCGTTC |
| EMacs6888-F | AGCCAGTGGCGATAAG AACTGGTGATGGCAGCAGGG |
| EMacs6888-R | AGCCAGTGGCGATAAG GAGGCTGCTGTCCTGCTCAC |
| EMppc4964-F | AGCCAGTGGCGATAAG CGTACCTACGGCACCGTAAG |
| EMppc4964-R | AGCCAGTGGCGATAAG GATGTCGCGCGCCACGATTG |
| EMacE3557F | AGCCAGTGGCGATAAG GTCCACAGCCTTGAGGCGCC |
| EMacE3557-R | AGCCAGTGGCGATAAG TCGGTGCCGAGCACCCGGTA |
| EMpyc3899-F | AGCCAGTGGCGATAAG TGCTTACCCGTTCGGCCTAG |
| EMpyc3899-R | AGCCAGTGGCGATAAG CTACCAGCACCTTGCGGAAC |
| EM pyk1865-F | AGCCAGTGGCGATAAG CTGAGCGAGATGGGCGATTC |
| EM pyk1865-R | AGCCAGTGGCGATAAG GTCGCCAGTGCCATGGAAAC |
| EMICL6749-F | AGCCAGTGGCGATAAG TGCCCTGACGTCGGAGACAC |
| EMICL6749-R | AGCCAGTGGCGATAAG TGCTGCCTGACTGAGCCATG |
|  |  |
|  |  |
| EMglpX2304-F | AGCCAGTGGCGATAAG GGACCGTCTGCCTTCCTCTC |
| EMglpX2304-R | AGCCAGTGGCGATAAG TGCTCGGTCATCAAGCGCAC |
| EMgap5957-F | AGCCAGTGGCGATAAG TGACCGAGGACCGTTCACGG |
| EMgap5957-R | AGCCAGTGGCGATAAG ATGCCTACGCGGATCGTCAC |
| EMpgi5962-F | AGCCAGTGGCGATAAG CGCTCTTCGCCCGTTGTACG |
| EMpgi5962-R | AGCCAGTGGCGATAAG GGTGAACACTGCTGAGGACC |
| EMpyk1865-F | AGCCAGTGGCGATAAG GACAAGGACCTCGTGGACGC |
| EMpyk1865-R | AGCCAGTGGCGATAAG AGCCGGTTCTTGCGCTGAAG |
| EMpyk5886-F | AGCCAGTGGCGATAAG TGACCGAGGACCGTTCACGG |
| EMpyk5886-R | AGCCAGTGGCGATAAG ATGCCTACGCGGATCGTCAC |

**Table S2.** **Transcriptome analysis of Differentially expressed genes (DEGs) between the mutant strain ΔrspA1 and the initial strain A30 in the EMP pathway and TCA pathway**

| Genes | Function | Log_2_(ΔrspA1/A30) |
| --- | --- | --- |
| EMP pathway | | |
| *SLNWT_3555* | Pyruvate dehydrogenase complex | -2.64 |
| *SLNWT_3556* |  | -2.79 |
| *SLNWT_3557* |  | -2.39 |
| *SLNWT_4515* |  | -0.51 |
| *SLNWT_4516* |  | -2.75 |
| *SLNWT_3542* |  | -2.44 |
| *SLNWT_3543* |  | -0.64 |
| *SLNWT_5957* | *gap* | -0.51 |
| Gluconeogenesis | | |
| *SLNWT_3899* | *pyc* | 0.91 |
| *SLNWT_4964* | *pck* | 0.96 |
| *SLNWT_4462* | *ppc* | -2.92 |
| *SLNWT_2304* | *glpX* | 1.22 |
| *SLNWT_5962* | *pgi* | 0.87 |
| TCA cycle | | |
| *SLNWT_5867* | *gltB* | -3.29 |
| *SLNWT_5868* | *gltD* | -3.80 |
| *SLNWT_2539* | *gdhA* | 2.37 |
| *SLNWT_2008* | *sucA* | 0.78 |
| *SLNWT_5701* |  | 0.72 |
| *SLNWT_4172* | *sdh* | 0.73 |
| *SLNWT_4173* |  | 1.04 |
| *SLNWT_4174* |  | 0.84 |
| *SLNWT_2307* | *fum* | 1.08 |
| *SLNWT_2312* |  | -0.19 |
|  |  |  |
| *SLNWT_5962* | *pgi* | 0.87 |
| *SLNWT_5964* | *pgl* | 0.45 |
| *SLNWT_5966* | *zwf* | 0.51 |
| *SLNWT_5967* | *tal2* | 0.53 |
| *SLNWT_5968* | *tkt* | 1.24 |
| *SLNWT_3348* | *argC* | -5.59 |
| *SLNWT_3349* | *argJ* | -5.10 |
| *SLNWT_3350* | *argB* | -4.89 |
| *SLNWT_3351* | *argD* | -5.01 |
| *SLNWT_6340* | *argC* | -0.75 |
| *SLNWT_6342* | *argJ* | -0.41 |
| *SLNWT_6343* | *argB* | -0.58 |
| *SLNWT_6344* | *argD* | -0.53 |
| ABC transporters |  |  |
| *SLNWT_4040* | *pstB* | -4.83 |
| *SLNWT_4041* | *pstA* | -4.58 |
| *SLNWT_4042* | *pstC* | -4.54 |
| *SLNWT_4043* | *pstS* | -5.22 |
| *SLNWT_1507* | *gluA* | -0.82 |
| *SLNWT_1508* | *gluB* | -0.58 |
| *SLNWT_1509* | *gluC* | -0.77 |
| *SLNWT_1510* | *gluD* | -0.63 |
